# Transmission dynamic of methicillin-resistant *Staphylococcus aureus* in a medical intensive care unit

**DOI:** 10.1186/1753-6561-5-S6-O73

**Published:** 2011-06-29

**Authors:** S Hugonnet, I Hall, I Barrass, S Leach, D Pittet

**Affiliations:** 1 University of Geneva Hospitals, Geneva, Switzerland; 2 Health Protection Agency, London, UK

## Introduction / objectives

Intensive care units (ICUs) play an important role in MRSA epidemiology. Although successful interventions are usually multimodal, the relative efficacy of single measures remains unknown. We developed a mathematical model to explore the transmission dynamic of MRSA and assess several control strategies.

## Methods

A discrete time individual-based stochastic model was built and calibrated on the number of crosstransmissions obtained through prospective surveillance. Most of the input parameters were derived from locally-acquired data. After model fitting and sensitivity analysis, several screening and isolation policies were tested by simulating the number of crosstransmissions and isolation-days under various scenarios.

## Results

The three unknown values were fitted to the model. Under the used assumptions, the environment played a negligible role. The number of crosstransmissions increased by almost 40% if only alert patients are screened and isolated, by about 50% if isolation is put in place only after the results of the admission screening become available, and over 60% in the absence of admission screening and isolation. The method used (culture or PCR) for admission screening had no impact on the number of crosstransmissions. Systematic regular screening during ICU stay provided no added-value.

## Conclusion

Aggressive admission screening and isolation are effective to reduce the number of crosstransmission. Colonized HCW may play an important role in MRSA transmission and HCW screening should be reinforced.

## Disclosure of interest

None declared.

